# Quantitative ultrasound evaluation of the thoracolumbar fascia after manual and acupuncture therapies: an exploratory mechanistic randomized controlled trial with a sequential within-subject phase

**DOI:** 10.1186/s41747-026-00754-7

**Published:** 2026-06-19

**Authors:** Norio Tomita, Dany Croteau, Marie-Hélène Roy-Cardinal, Manon Choinière, Laura Benhaïm, Sara Raby, Aline Boulanger, Jill Runquist, Nathaly Gaudreault, Guy Cloutier, Nathalie J. Bureau

**Affiliations:** 1 Laboratory of Biorheology and Medical Ultrasonics, Research Center, Centre hospitalier de l’Université de Montréal (CHUM), Montreal, QC Canada; 2 Institute of Biomedical Engineering, Université de Montréal, Montreal, QC Canada; 3 Faculty of Medicine, Université de Montréal, Montreal, QC Canada; 4 Department of Anesthesiology and Pain Medicine, Université de Montréal, Montreal, QC Canada; 5 Research Center, CHUM, Montreal, QC Canada; 6 Le CocOOOn Thérapeutique, Montreal, QC Canada; 7 La Lignée, Clinique d’acupuncture, Sara Raby, Ac–propriétaire, Montreal, QC Canada; 8 Pain Clinic, CHUM, Montreal, QC Canada; 9 HOMAthérapie, Montreal, QC Canada; 10 Faculty of Medicine and Health Sciences, Université de Sherbrooke, Sherbrooke, QC Canada; 11 Research Center, Centre hospitalier Universitaire de Sherbrooke, Sherbrooke, QC Canada; 12 Department of Radiology, Radio-oncology and Nuclear Medicine, Université de Montréal, Montreal, QC Canada

**Keywords:** Biomechanical phenomena, Complementary therapies, Fascia, Low back pain, Ultrasonography

## Abstract

**Objective:**

Thoracolumbar fascia (TLF) alterations may contribute to nonspecific low back pain (NSLBP). We evaluated the effects of acupuncture, chiropractic care, and massage on TLF biomechanics, microstructure, and clinical outcomes in NSLBP.

**Materials and methods:**

In this exploratory mechanistic hybrid trial, sixty participants (median age: 47 years [interquartile range 39–63]; 63% women) were randomized (1:1:1) to acupuncture, chiropractic, or a 3-week waitlist control, providing between-group comparisons. After the waitlist, control participants received massage therapy, enabling a sequential within-subject evaluation. Pre- and post-intervention assessments included TLF shear strain (ShS)—cumulated (C|ShS|_L_) and maximum (Max|ShS|_L_) absolute lateral strain—homodyned K-distribution (HKD) parameters, and clinical scores. Analyses used linear mixed-effects models, Fisher’s exact test, and Spearman’s rank correlation.

**Results:**

In the randomized comparison, C|ShS|_L_ decreased with chiropractic (*β*: -16%, 95% confidence interval [-29, -2]; *p* = 0.021; *d* = -0.322), but not with acupuncture (*p* = 0.130), and increased in controls (*β*: +19%, *p* = 0.003; *d* = +0.387), with a smaller rise in Max|ShS|_L_ (*β*: +0.4%, *p* = 0.048). Massage following the control phase reduced C|ShS|_L_ (*β*: -32%, *p* < 0.001; *d* = -0.673) and Max|ShS|_L_ (*β*: -0.5%, *p* = 0.010). The control–massage phase interaction effect size for C|ShS|_L_ was large (*d* = 1.082). HKD parameters remained unchanged. Only chiropractic yielded improvement in the Oswestry disability index (ODI) (*p* = 0.041). No correlation was observed between changes in C|ShS|_L_ and ODI (ρ = 0.261; *p* = 0.054).

**Conclusion:**

Chiropractic and massage, unlike acupuncture, elicited detectable biomechanical changes, while chiropractic also enhanced disability outcomes. HKD detected no microstructural changes.

**Trial registration:**

Clinicaltrials.gov, NCT04843800. Registered April 14, 2021 (https://clinicaltrials.gov/study/NCT04843800).

**Relevance statement:**

This study shows that ultrasound-derived shear strain captures the short-term mechanical responses of the thoracolumbar fascia to chiropractic care or massage therapy in participants with chronic nonspecific low back pain, supporting the potential role of quantitative fascial imaging biomarkers for mechanistic assessment and treatment monitoring of this prevalent, debilitating disorder.

**Key Points:**

- Recent research suggests that altered microstructure and biomechanics of the thoracolumbar fascia may contribute to nonspecific low back pain.
- In this randomized trial, chiropractic care, but not acupuncture, reduced thoracolumbar shear strain, while controls showed increased strain.
- In the within-subject sequential analysis, thoracolumbar shear strain increased during the waitlist phase and decreased after massage therapy.
- No microstructural changes were detected over 3 weeks, potentially reflecting biological constraints and/or limitations in measurement sensitivity.
- Small sample size and low baseline disability likely limited the trial’s power to detect significant effects on clinical outcomes.

**Graphical Abstract:**

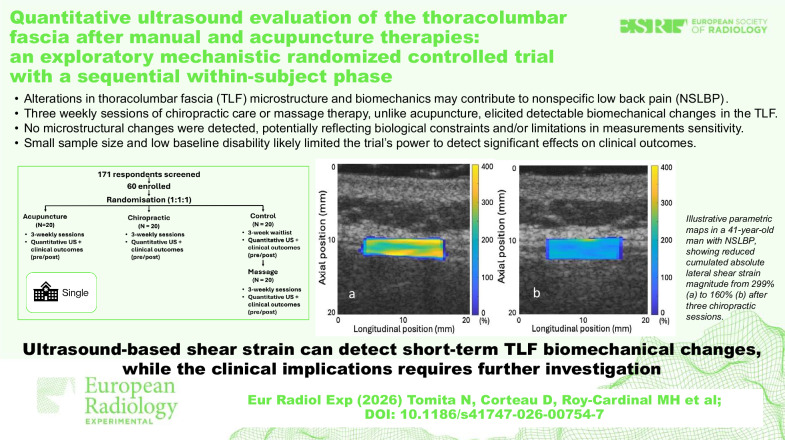

## Background

Low back pain is the leading cause of disability worldwide [1]. When no specific cause is identified, it is classified as nonspecific low back pain (NSLBP), representing approximately 90% of all cases [2, 3].

The thoracolumbar fascia (TLF) is richly innervated [4, 5] and prone to structural and inflammatory changes [6], supporting its involvement in NSLBP pathophysiology [6, 7]. A proposed model suggests that an initial fascial injury, compounded by restricted movement due to stiffness, pain, or fear of pain, sustains inflammation and leads to fibrosis, adhesions, and impaired TLF mobility [8]. Experimental data support this mechanism: in a porcine model, combined fascial injury and movement restriction increased TLF thickness and reduced sliding after 8 weeks [9].

Nonpharmacologic interventions—such as acupuncture, multimodal chiropractic care, and massage—have shown clinical benefits in NSLBP [10]. Their effects may, in part, involve modulation of fascial properties [11, 12]. Acupuncture provides short-term pain relief [13], mobilizes connective tissue as shown on ultrasound [14], and acts at fascia-rich interfaces [15], supporting a plausible effect on the TLF. Mechanistically, needling stretches connective tissue and induces remodeling through mechanotransduction [16–18]. Chiropractic also reduces pain and disability [19], primarily *via* neurophysiological modulation of pain pathways [20], with recent ultrasound data suggesting improved TLF mobility [21]. Massage improves pain and disability [22], possibly by enhancing fascial mobility [23], reducing stiffness [24], or triggering neurophysiological responses [25], though the evidence is inconsistent [26, 27].

To determine whether these therapies directly affect fascia, reliable quantitative markers of TLF alterations are necessary. We previously demonstrated in a case-control study that patients with NSLBP exhibit increased TLF shear strain (ShS), interpreted as abnormal tension and impaired sublayer sliding [28]. In the same cohort, altered microstructural properties, suggestive of fibrosis and viscosity, were detected using quantitative ultrasound (QUS) with homodyned K-distribution (HKD) statistical modeling [29].

In this exploratory mechanistic hybrid trial, participants were randomized to acupuncture, chiropractic care, or a waitlist control to evaluate effects on TLF properties. After the waitlist phase, control participants received massage therapy, enabling a within-subject sequential analysis. The primary outcome was TLF ShS; secondary outcomes included QUS HKD metrics and patient-reported clinical outcomes. Based on our previous studies [28, 29], we hypothesized that all active treatments would reduce ShS, increase HDK metrics, and improve clinical outcomes relative to the control group.

## Methods

This single-center trial was approved by our institution’s ethics committee (CE 21.003) and was registered on Clinicaltrials.gov on April 14, 2021 (https://clinicaltrials.gov/study/NCT04843800).

### Recruitment of participants

Participants were recruited through advertisements in the “Quebec Chronic Low Back Pain Registry” newsletter, social media platforms, and the hospital’s television information system.

During the pre-registration phone call, the research assistant screened respondents, provided an overview of the study, and assessed their eligibility. The principal investigator (N.J.B.) validated participants’ eligibility before the research visit.

Inclusion criteria encompassed individuals aged 18 to 75 years who were experiencing persistent NSLBP or referred pain above or just below the gluteal fold. Participants had to report a pain intensity of at least 3 out of 10 on a numerical pain rating scale, with symptoms lasting at least 3 months and occurring on at least 50% of days in the preceding 6 months [30]. Participants were required to abstain from initiating new treatments during the study. They could continue taking their usual medication to manage their low back pain during the 4 weeks before and during the study. Exclusion criteria are listed in Table 1.

**Table 1:** Exclusion criteria

Category	Specific conditions
Specific pathologies	Intervertebral disc herniation with neurological symptoms, radicular pain (sciatica), infectious spondylodiscitis, low back pain due to inflammatory, malignant, or autoimmune diseases, congenital spinal deformities (excluding slight scoliosis), compression fractures due to osteoporosis, spinal stenosis, spondylolysis, or spondylolisthesis.
Pregnancy	Pregnant individuals were excluded.
Surgical or trauma history	History of spinal surgery or pelvis/hip fractures.
Recent therapeutic interventions	Received acupuncture, spinal manipulation, or therapeutic massage for back pain within the past year.
Hematologic conditions	Severe clotting disorders or ongoing anticoagulant therapy.

### Enrollment visits and baseline ultrasound

The research assistant invited eligible participants to review and sign the information and consent form and complete self-administered questionnaires. Demographic information was collected, and participants’ weight, height, and lumbar spine flexion range—assessed using the Schober test [31]—were recorded. The normative value for lumbar spine flexion is 6.85 ± 1.18 cm (mean ± standard deviation) [32].

All ultrasound examinations were performed by a fellowship-trained musculoskeletal radiologist with more than 25 years of experience (N.J.B.), blinded to group allocation. Baseline scans were acquired before randomization, and follow-up scans were conducted without access to participant assignment or intervention status. Radiofrequency data acquisition was performed using a Terason T3000 ultrasound scanner (v4.7.7, Terason Ultrasound) equipped with a 12L5 linear array transducer. Ultrasound data were acquired according to a previously established protocol [28]. Participants were positioned prone on a motorized table (Echo Flex Model 4,800, Ibiom Instruments) with their iliac crests aligned with the hinge, allowing passive flexion of the lower limbs at a constant, reproducible speed. The transducer was placed on the participant’s lower back, laterally at the L2–L3 level, over the *erector spinae* muscles’ apex, and scanning was carried out in the longitudinal plane. Three consecutive 10-s radiofrequency ultrasound recordings were taken on each side of the spine (6 acquisitions per participant) during passive flexion and extension of the lower limbs, as the table tilted downward by 20 degrees and then returned to its neutral position. Each recording consisted of a 4-s stationary phase, a 3-s downward tilt, and a 3-s upward return. Imaging and focus depths were adjusted based on participants’ body habitus, ranging from 2.0 to 5.0 cm and 1.3 to 3.5 cm, respectively. The frequency was set to “middle,” and the frame rate ranged from 22.4 to 59.8 frames/s.

### Randomization

After baseline assessment, participants were randomly assigned in a 1:1:1 ratio to acupuncture, chiropractic care, or a waitlist control in a parallel-group design, providing between-group comparisons. After the 3-week waitlist period, participants initially allocated to the control group received massage therapy; this sequential phase was analyzed separately using a within-subject model to evaluate mechanistic responsiveness.

Randomization used a computer-generated block sequence (size six) prepared by an independent biostatistician. Allocations were concealed in sequentially numbered sealed envelopes managed by the research assistant.

### Interventions

All active interventions were delivered once weekly for three consecutive weeks. Session durations were 45 min for acupuncture and massage, and 20 min for chiropractic care. The acupuncture protocol combined Western physiological and Eastern traditional approaches to release myofascial tension and modulate autonomic function. Chiropractic care was multimodal, incorporating spinal manipulation, mobilization, and soft tissue therapy. Massage focused on myofascial release techniques targeting the TLF and related fascial chains. Certified practitioners delivered the standardized treatments—acupuncture (S.R., J.R.), chiropractic (L.B.), or massage (N.T.)—at their respective clinics. Treatment protocols are detailed in the Supplementary material (Supplementary material: Treatment protocols).

### Outcome measures and time points

All outcomes were assessed at baseline, at week 4, following the waiting period for the control group, and at week 4 after treatment for the acupuncture, chiropractic, and massage groups.

**Fig. 1:**
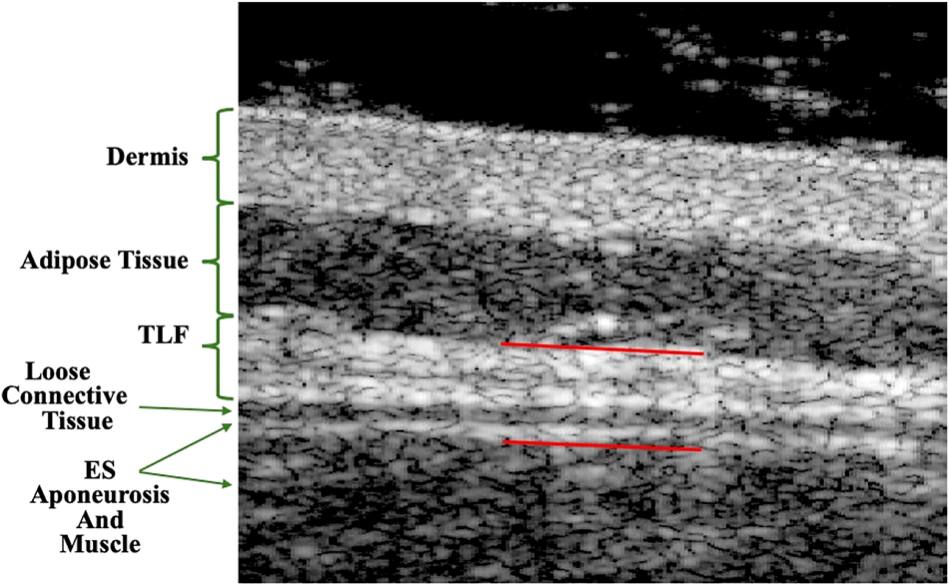
Ultrasound anatomy of the paraspinal soft tissues and definition of the region of interest (ROI) for radiofrequency data processing. A longitudinal (long-axis) B-mode image of the paraspinal soft tissues in a 77-year-old male with nonspecific low back pain shows the distinct anatomical layers: dermis, subcutaneous adipose tissue, multilaminar thoracolumbar fascia (TLF), the hypoechoic hyaluronan-rich loose connective tissue layer, and the hyperechoic aponeurosis and muscle of the *erector spinae* (ES). The ROI includes pixels within the two red 10-mm lines, drawn at the superficial TLF and the deep ES aponeurosis

The primary outcome was TLF ShS. ShS quantifies angular deformation induced by shearing forces. Pixel displacements within the image region of interest (ROI) were tracked to capture shape changes and relative motion between the TLF sublayers and the *erector spinae* aponeurosis during passive flexion of the lower limbs. Values near zero indicate no shearing motion or uniform translation in a single direction of imaged structures, whereas larger absolute values reflect shearing occurring in multiple directions. Radiofrequency datasets were analyzed using custom C++ and MATLAB (vR2024a, Mathworks Inc.) programs. ROI delineation and image processing were performed on anonymized datasets by a master’s student (DC), who was blinded to participant identity, group allocation, and time point. The ROI was defined as a 10-mm horizontal line spanning the superficial TLF and the deep border of the *erector spinae* aponeurosis on the central frame of the B-mode cine loop acquired during table-downward tilt [28] (Fig. 1). Although both downward and upward motion phases were recorded, only the downward (flexion) phase was analyzed, as previous work [28] found no significant difference in ShS between phases. This approach reduced redundancy while maintaining physiological relevance. The ROI was then automatically propagated across frames using motion compensation, and ShS was computed with the Lagrangian speckle model estimator [33]. Two parameters were extracted: the averaged cumulated absolute lateral ShS magnitude (C|ShS|_L_) and the maximum absolute lateral ShS (Max|ShS|_L_). This method has demonstrated excellent intra-operator reliability [28].

Secondary outcomes included QUS HKD parameters along with pain and disability scores [30].

When ultrasound propagates through biological tissues, it encounters microstructural components with varying acoustic impedances, producing scattered waves that interfere and form a speckle pattern. This pattern reflects microstructural features of the tissue, including cell size, cell packing, and spatial organization [34–36]. HKD statistical analysis of the radiofrequency signal envelope was applied to characterize these features. For HKD estimation, the same ROI was defined at the center of the first frame of the reconstructed B-mode cine loop during the initial stationary phase, thereby reducing motion-related artifacts. Radiofrequency datasets were analyzed with a MATLAB custom software to compute: (1) 1/α, the reciprocal of the scattering cluster parameter (scatterer density), and (2) 1/(κ + 1), the diffuse-to-total signal power ratio (scatterer organization). Values were averaged over the first 10 stationary-phase frames [29]. This method provides accurate ROI delineation, with good interrater reliability for 1/α and moderate reliability for 1/(κ + 1) [29].

Patient-reported outcomes were self-administered *via* a secure online platform in the presence of the research assistant, who was not involved in ultrasound data analysis. Questionnaires included the Brief Pain Inventory (BPI) Short Form [37], the Oswestry disability index (ODI) [38, 39], and the Patient-Reported Outcomes Measurement Information System (PROMIS) Short Form v1.1–Global Health [40]. The BPI assessed average pain intensity over the past 24 h, and pain interference (0–10 scale; higher = worse). The ODI measured disability on a 0–100% scale, categorized as minimal (0–20%), moderate (21–40%), severe (41–60%), crippled (61–80%), or bedbound (81–100%). PROMIS provided standardized Physical and Mental Health T-scores (50 ± 10, mean ± standard deviation, in the U.S. general population), with values below 50 indicating below-average function or health.

### Sample size calculation

Because no prior data were available for ShS (the primary outcome) at the time the study protocol was registered, the sample size calculation was based on the ODI, a well-established measure of the clinical efficacy of therapeutic interventions [41]. The ODI has a minimal clinically important difference (MCID) of 10 points [39], which was chosen as the threshold for detecting meaningful within-group improvements at 4 weeks compared to baseline, following waitlist or therapy. Using this criterion, a sample size of 81 patients (27 per intervention arm) was required to provide 80% power, assuming a baseline mean ODI score of 29 ± 20 (mean ± standard deviation), as reported in previous studies [42, 43]. Although ShS was the designated primary outcome, the ODI was selected as a surrogate for sample size estimation because it reflects clinically meaningful functional improvements. We reasoned that measurable changes in the TLF’s mechanical and microstructural characteristics would likely accompany evidence of clinical efficacy, as reflected in ODI scores. To account for an anticipated 19% attrition rate, the final target sample size was set at 96 patients.

### Statistical analyses

Shapiro–Wilk tests indicated non-normal distributions. To maintain consistency and facilitate interpretation, analyses were conducted on untransformed data, as square-root transformation yielded identical statistical conclusions. Model assumptions (residual normality and homoscedasticity) were satisfied, and the known robustness of linear mixed-effects models supported this approach.

Longitudinal changes in ultrasound outcomes were analyzed separately for the randomized parallel-group component and the sequential within-subject phase using linear mixed-effects models.

For the randomized comparison (acupuncture, chiropractic care, waitlist control), models included fixed effects for Group, Time (pre *versus* post), and their interaction, with a participant-specific random intercept. Least-squares means and 95% confidence intervals (CIs) were estimated, and interaction contrasts were used to derive between-group differences in change.

For the sequential phase, analyses were restricted to participants initially assigned to a waitlist control who subsequently received massage therapy. Models included fixed effects for Phase (waitlist *versus* massage), Time, and their interaction, with a random intercept. This approach tested whether pre–post changes during massage differed from those observed during the waitlist phase, thereby assessing mechanistic responsiveness independently of the randomized comparison.

Frame rate (for ShS) and ROI depth (for QUS HKD parameters) were added as covariates to the models because of their known influence on these outcomes [35, 36]. *Post hoc* pairwise comparisons were Bonferroni-adjusted. Secondary outcomes and *post hoc* contrasts were considered exploratory. Analyses followed an intention-to-treat approach, including all randomized participants with at least one baseline imaging outcome. Sensitivity analyses used a per-protocol dataset, excluding participants with missing outcomes. To assess potential period effects inherent to the sequential design, a sensitivity analysis was performed using an alternative mixed-effects model that treated the three time points (T_0_, T_1_, T_2_) as categorical levels and adjusted for visit order.

Fisher’s exact test compared the proportion of participants meeting the MCID thresholds: ≥ 10% reduction in BPI average pain, ≥ 1-point reduction in BPI interference, ≥ 12% improvement in ODI, and ≥ 3-point improvement in PROMIS Physical and Mental T-scores.

To explore the relationship between imaging and clinical outcomes, we conducted an exploratory correlation analysis between changes in C|ShS|_L_ and ODI scores (Δ = Post *minus* Pre) among participants in the intervention groups (acupuncture, chiropractic, and massage; *n* = 55), using Spearman’s rank correlation.

Statistical analyses were performed by one author (DC) using R version 4.2.1 (The R Foundation for Statistical Computing) and independently verified by a biostatistician. All tests were two-tailed, with statistical significance set at *p* < 0.05.

## Results

### Participants

**Fig. 2:**
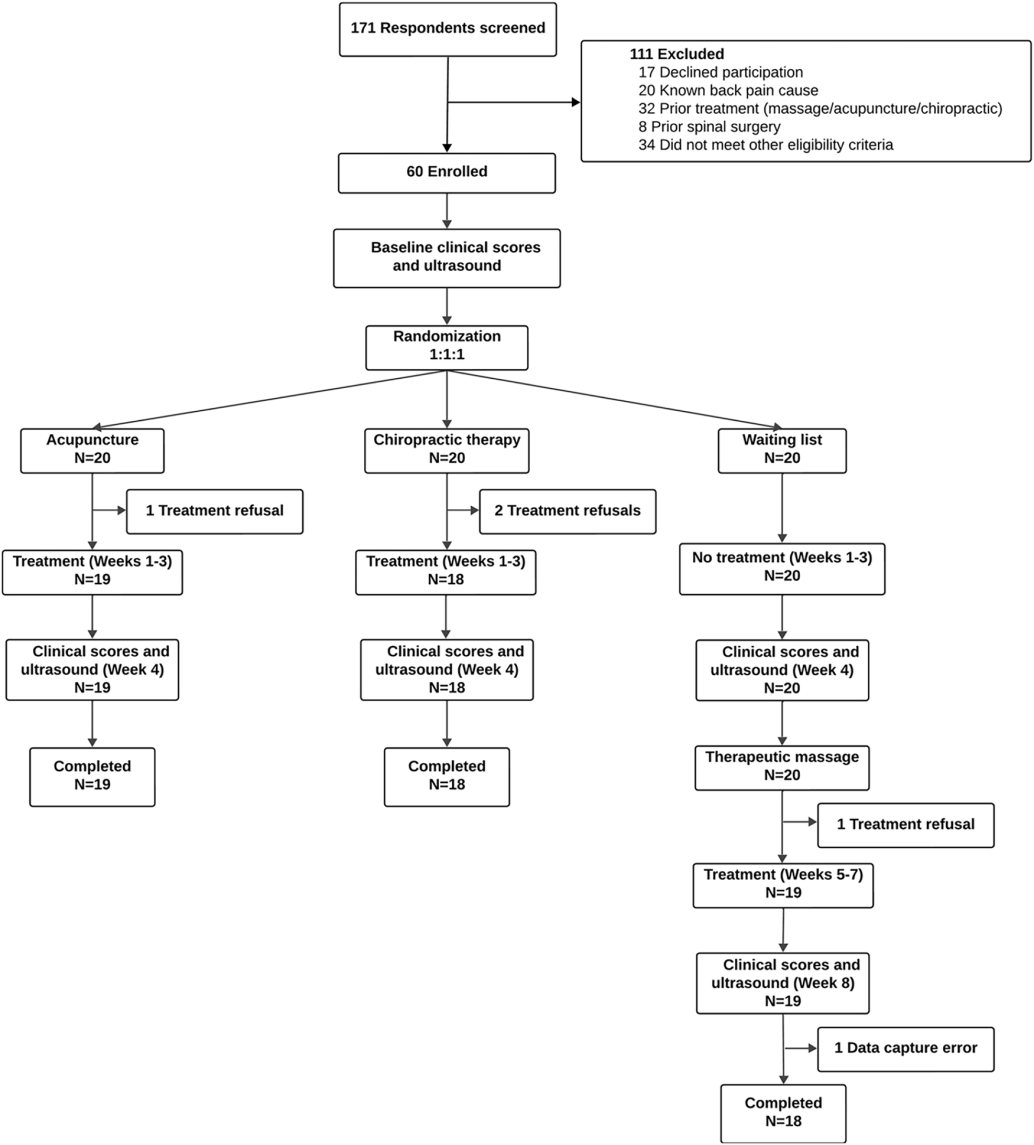
Trial enrollment and follow-up chart. Sixty participants were randomly assigned (1:1:1) to acupuncture, chiropractic or a 3-week waitlist control. After the waitlist, control participants received massage therapy. Assessments were performed at baseline and at week 4, following the waiting period (control group) and post-therapy (acupuncture, chiropractic, and massage groups)

Between November 2022 and July 2024, the research assistant screened 171 respondents. Due to funding limitations, enrollment was terminated early, resulting in 60 participants (median age, 47 years [interquartile range, 39–63]; 38 (63%) women). One participant from each massage and acupuncture group, and two from the chiropractic group, withdrew after declining their assigned therapy. Additionally, one data recording error occurred in the massage group. Thus, the final analysis included data from 55 participants. No adverse events or deviations from medication stability requirements or restrictions on co-interventions were reported during the trial. Figure 2 shows the trial enrollment and follow-up process.

Baseline characteristics (Table 2) were comparable across the randomized groups (acupuncture, chiropractic, and waitlist control) and the post-waitlist massage group. Notably, for psychological status, median PROMIS mental health T-scores ranged from 45.8 [interquartile range 42.3–49.6] to 50.8 [45.2–53.3] across groups, consistent with general USA population norms (50 ± 10, mean ± standard deviation), indicating the absence of marked psychological distress within the cohort.

**Table 2:** Baseline demographics, ultrasound parameters, and clinical characteristics

Variable		Groups			
		Waitlist control^a^	Post-waitlist massage^a^	Acupuncture	Chiropractic
Number		20	20	20	20
Sex, *n* (%)	Men	7 (35.0)	7 (35.0)	7 (35.0)	8 (40.0)
	Women	13 (65.0)	13 (65.0)	13 (65.0)	12 (60.0)
Age (years)		45.0 [38.8–63.8]	45.0 [38.8–63.8]	43.5 [38.8–50.3]	51.0 [43.3–63.8]
BMI (kg/m^2^)		26.7 [24.4–31.0]	26.7 [24.4–31.0]	27.5 [24.7–30.5]	27.0 [24.9–29.1]
C|ShS|_L_ (%)		205 [154–278]	228 [149–303]	218 [175–259]	222 [167–284]
Max|ShS|_L_ (%)		5.0 [4.0–6.2]	5.6 [4.0–7.1]	5.2 [4.3–6.3]	5.2 [4.2–7.0]
1/*α* (unitless)		0.73 [0.57–0.85]	0.73 [0.60–0.87]	0.74 [0.64–0.91]	0.73 [0.62–0.86]
1/(*κ* + 1) (unitless)		0.34 [0.29–0.39]	0.31 [0.28–0.36]	0.33 [0.30–0.39]	0.32 [0.28–0.36]
Schober (cm)		5.5 [4.4–6.5]	5.0 [4.0–6.0]	5.5 [4.9–6.8]	5.3 [4.4–6.6]
BPI average pain (24 h)		3.0 [3.0–4.0]	3.0 [2.0–4.0]	4.0 [2.8–6.0]	4.0 [2.0–5.0]
BPI interference		1.0 [0.4–2.7]	1.2 [0.4–1.9]	2.1 [1.0–3.9]	2.5 [1.4–3.5]
ODI, (%)		17.0 [11.5–20.5]	16.0 [8.0–24.0]	19.0 [13.0–27.0]	24.0 [15.5–29.5]
PROMIS Physical T-score		42.3 [39.2–47.7]	43.6 [39.8–50.8]	42.3 [39.2–44.9]	41.1 [37.4–44.9]
PROMIS Mental T-score		50.8 [45.2–53.3]	47.1 [43.5–53.3]	45.8 [42.3–49.6]	48.3 [42.9–50.8]

1/α = the reciprocal of the scatterer clustering parameter (scatterer density), 1/(κ + 1) = the diffuse-to-total signal power ratio parameter (scatterer organization)

*BMI* Body mass index, *BPI* Brief pain inventory, *C|ShS|*_*L*_ Cumulated absolute lateral shear strain magnitude, *Max|ShS|*_*L*_ Maximum absolute lateral shear strain, *ODI* Oswestry disability index, *PROMIS* Patient-Reported Outcomes Measurement Information System

^a^ The waitlist control and post-waitlist massage groups included the same participants. For the waitlist control group, the baseline assessment was performed after randomization; for the post-waitlist massage group, it was performed at week 4, following a 3-week waitlist period. Continuous variables are presented as median [interquartile range], while categorical variables are frequency (percentage)

However, the study population exhibited relatively mild baseline disability, with median ODI scores ranging from 17.0 [interquartile range 11.5–20.5] to 24.0 [15.5–29.5] across groups. These values were lower than anticipated in the sample size calculation, which assumed a baseline ODI score of 29 ± 20 (mean ± standard deviation).

### Effects of treatments on the primary outcome (TLF ShS—mechanical behavior)

#### Randomized parallel-group analysis

**Fig. 3:**
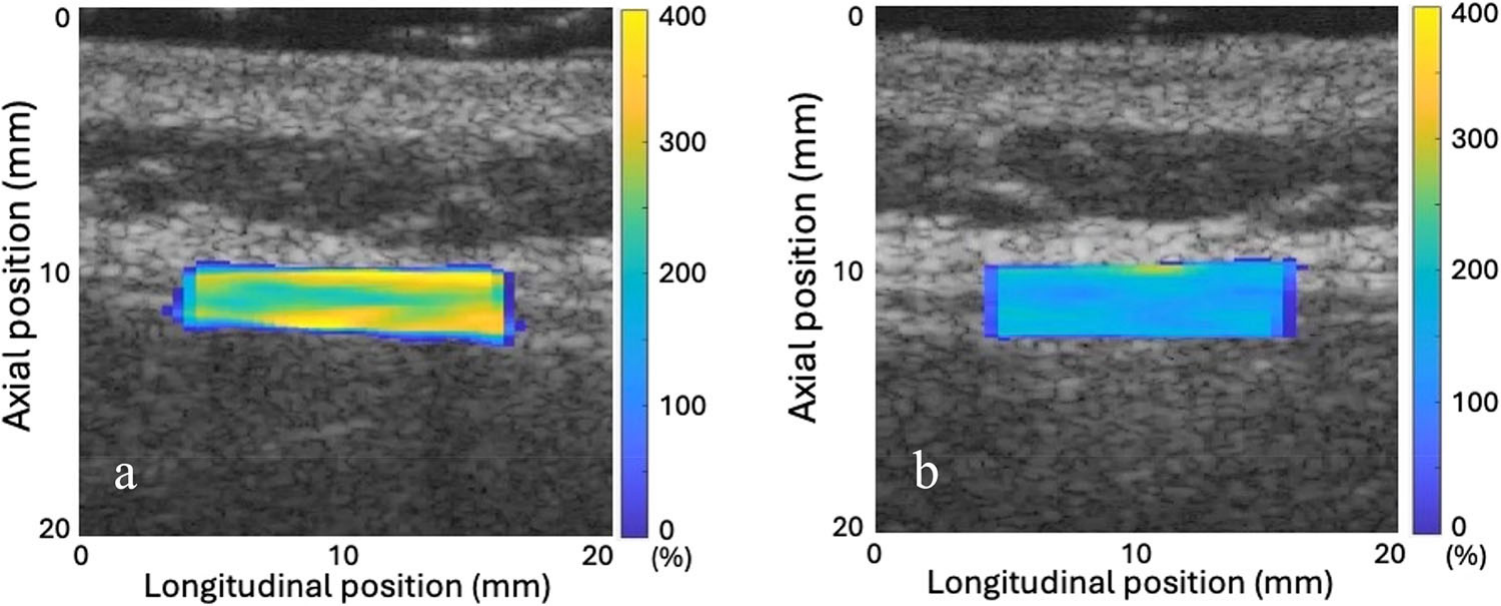
Parametric maps of shear strain in the thoracolumbar fascia in a 41-year-old man with nonspecific low back pain. Parametric maps illustrate changes in the cumulated absolute lateral shear strain magnitude (C|ShS|_L_) before (**a**) and after (**b**) three chiropractic care sessions, revealing a post-treatment reduction from 299% to 160%

**Fig. 4:**
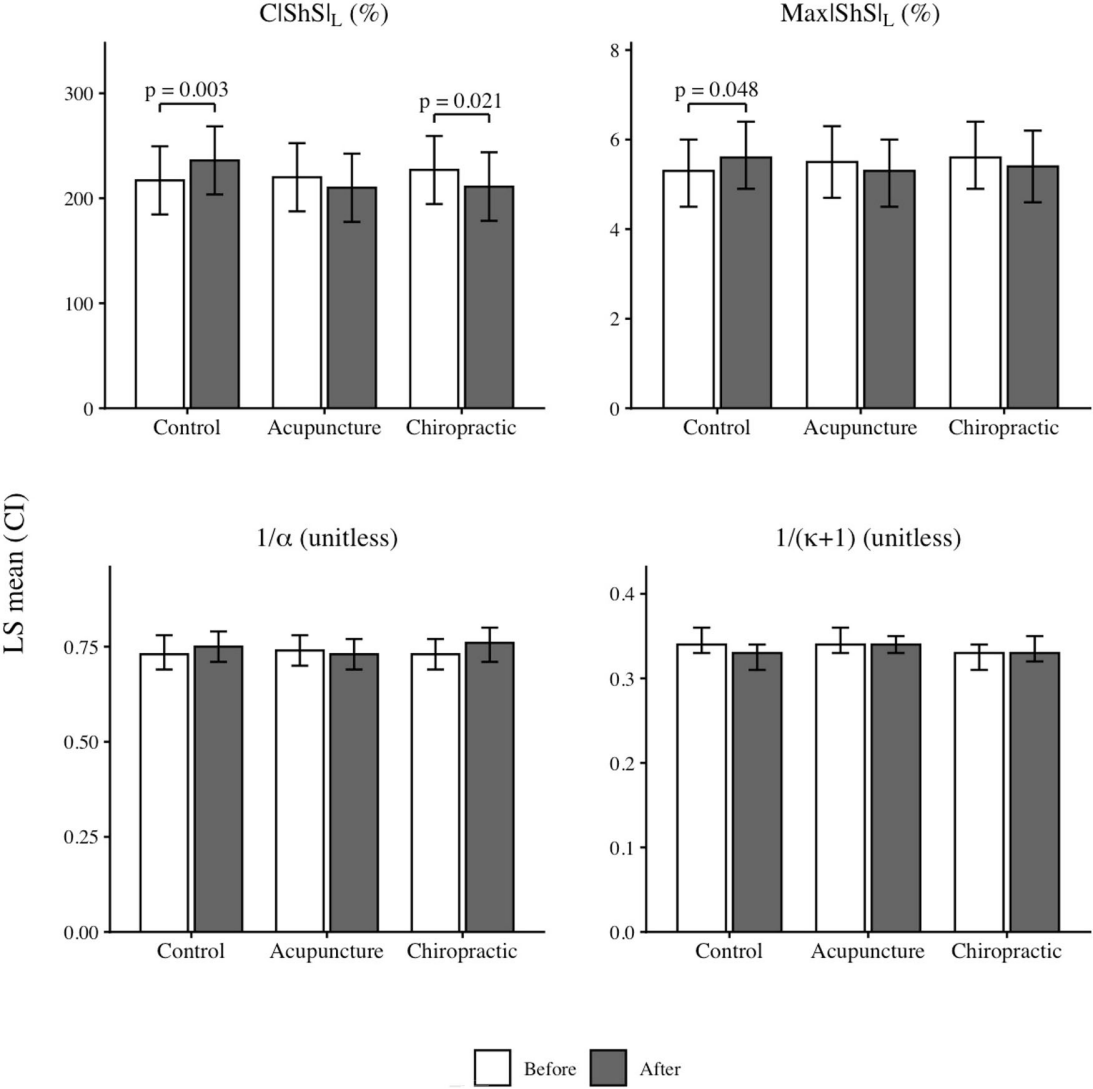
Estimated within-group changes in ultrasound outcomes in the randomized parallel groups (intention-to-treat analysis). The graphs display the estimated marginal means (least-squares means) ± 95% confidence intervals for the three randomized groups (control, acupuncture, chiropractic) at baseline and week 4. 95% CI, 95% Confidence interval; C|ShS|_L_, Cumulated absolute lateral shear strain magnitude; LS, Least squares; Max|ShS|_L_, Maximum absolute lateral shear strain. 1/α = the reciprocal of the scatterer clustering parameter (scatterer density), and 1/(κ + 1) = the diffuse-to-total signal power ratio parameter (scatterer organization)

A significant within-group reduction in C|ShS|_L_ was observed in the chiropractic group (*β*: -16%, 95% CI [-29, -2], *p* = 0.021; *d* = -0.322) (Fig. 3), whereas no significant change was detected in the acupuncture group (*β*: -10%, 95% CI [-23, 3], *p* = 0.130). In contrast, the control group demonstrated a significant increase in C|ShS|_L_ over time (*β*: +19%, 95% CI [7, 31], *p* = 0.003; *d* = 0.387) (Table 3) (Fig. 4). Between-group comparisons showed statistically significant differences in C|ShS|_L_ between control and acupuncture (*β*: +29%, 95% CI [7, 51], *p* = 0.005; *d* = 0.591), and between control and chiropractic (*β*: +35%, 95% CI [12, 57], *p* = 0.001; *d* = 0.709), but not between acupuncture and chiropractic (*β*: +6%, 95% CI [-17, 28], *p* = 1.000) (Supplementary Table S1). For Max|ShS|_L_, the control group showed a statistically significant increase (*β*: +0.4%, 95% CI [0.004, 0.7], *p* = 0.048; *d* = 0.257), with no significant changes observed in the acupuncture (*β*: -0.2%, *p* = 0.244) or chiropractic (*β*: -0.2%, *p* = 0.227) groups. Between-group differences in Max|ShS|_L_ were not statistically significant.

**Table 3:** Linear mixed-effects model estimates of randomized parallel-group treatment effects on ultrasound outcomes (intention-to-treat analysis)

Parameter	Group	Before LS mean [95% CI]	After LS mean [95% CI]	Adjusted change estimate (*β*) [95% CI]	*p*-value	Effect size (*d*)
C|ShS|_L_ (%)	Control	217 [185, 250]	236 [204, 269]	19 [7, 31]	0.003	0.387
	Acupuncture	220 [188, 253]	210 [178, 243]	-10 [-23, 3]	0.130	-0.204
	Chiropractic	227 [195, 259]	211 [179, 244]	-16 [-29, -2]	0.021	-0.322
Max|ShS|_L_ (%)	Control	5.3 [4.5, 6.0]	5.6 [4.9, 6.4]	0.4 [0.004, 0.7]	0.048	0.257
	Acupuncture	5.5 [4.7, 6.3]	5.3 [4.5, 6.0]	-0.2 [-0.6, 0.2]	0.244	-0.157
	Chiropractic	5.6 [4.9, 6.4]	5.4 [4.6, 6.2]	-0.2 [-0.6, 0.2]	0.227	-0.167
1/α (unitless)	Control	0.73 [0.69, 0.78]	0.75 [0.71, 0.79]	0.01 [-0.02, 0.05]	0.479	0.092
	Acupuncture	0.74 [0.70, 0.78]	0.73 [0.69, 0.77]	-0.01 [-0.05, 0.03]	0.551	-0.079
	Chiropractic	0.73 [0.69, 0.77]	0.76 [0.71, 0.80]	0.03 [-0.01, 0.07]	0.145	0.197
1/(κ + 1) (unitless)	Control	0.34 [0.33, 0.36]	0.33 [0.31, 0.34]	-0.02 [-0.03, 0.001]	0.068	-0.236
	Acupuncture	0.34 [0.33, 0.36]	0.34 [0.33, 0.35]	-0.003 [-0.02, 0.02]	0.743	-0.043
	Chiropractic	0.33 [0.31, 0.34]	0.33 [0.32, 0.35]	0.01 [-0.01, 0.02]	0.563	0.077

1/α = the reciprocal of the scatterer clustering parameter (scatterer density). *β* = percentage change relative to the reference group mean. 1/(κ + 1) = the diffuse-to-total signal power ratio parameter (scatterer organization)

*CI* Confidence interval, *C*|*ShS*|_*L*_ Cumulated absolute lateral shear strain magnitude, *d* Cohen *d* effect size, *LS* Least-squares mean, *Max*|*ShS*|_*L*_ Maximum absolute lateral shear strain

#### Sequential within-subject massage analysis

**Fig. 5:**
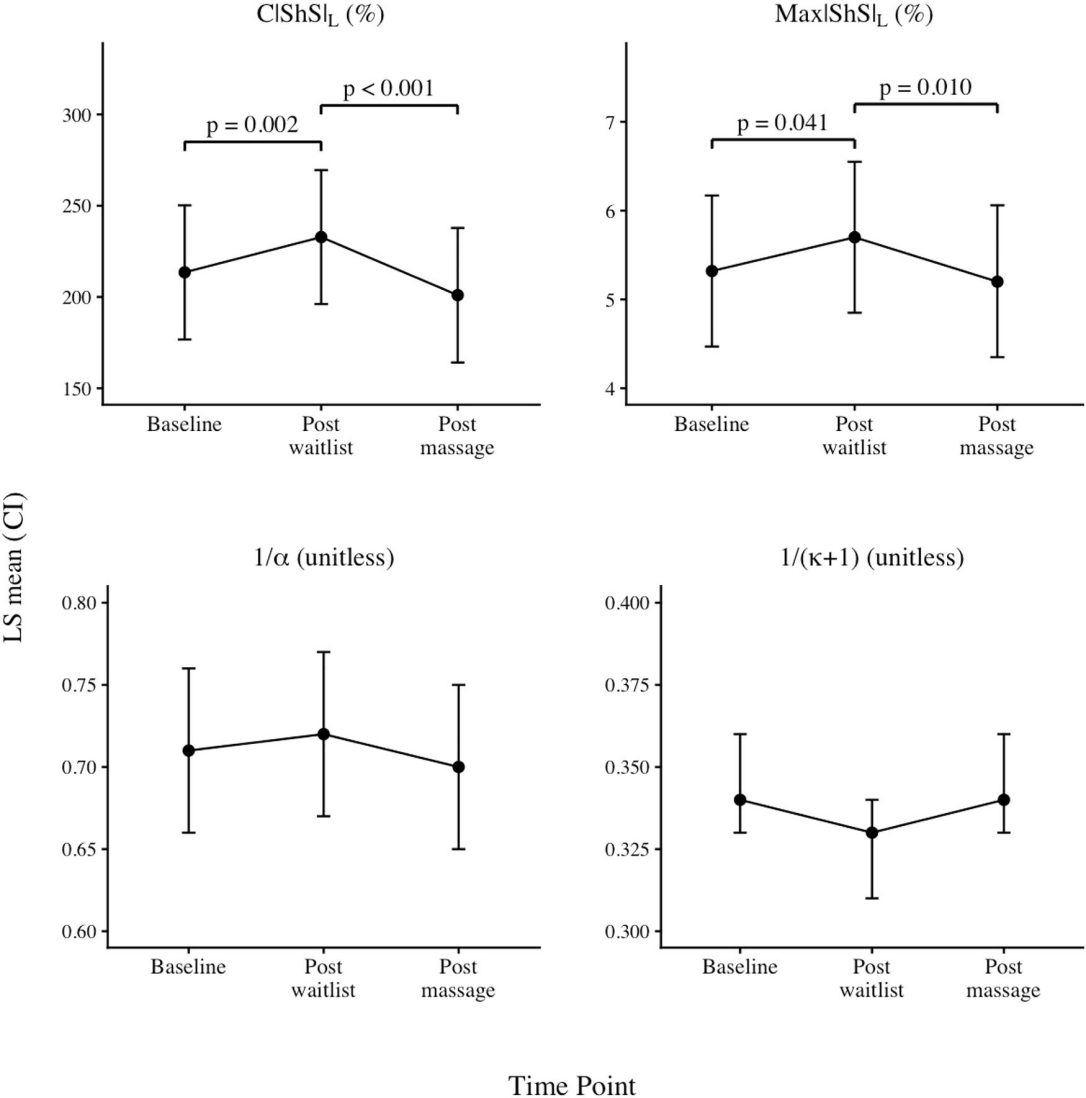
Estimated within-subject changes in ultrasound outcomes during the waitlist and massage phases (intention-to-treat analysis). The graphs display the estimated marginal means (least-squares means) ± 95% confidence intervals. 95% CI, 95% Confidence interval; C|ShS|_L_, Cumulated absolute lateral shear strain magnitude; LS, Least squares; Max|ShS|_L_, Maximum absolute lateral shear strain. 1/α = the reciprocal of the scatterer clustering parameter (scatterer density). 1/(κ + 1) = the diffuse-to-total signal power ratio parameter (scatterer organization)

Among participants who received massage following the waitlist control phase, C|ShS|_L_ (*β*: -32%, 95% CI [-44, -19]; *p* < 0.001; *d* = -0.673), and Max|ShS|_L_ (*β*: -0.5%, 95% CI [-0.9, -0.1]; *p* = 0.010; *d* = -0.346) significantly decreased. In contrast, both parameters increased during the preceding control phase (C|ShS|_L_: *β*: +19%, 95% CI [7, 31]; *p* = 0.002; *d* = 0.409; Max|ShS|_L_: *β*: +0.4%, 95% CI [0.02, 0.8]; *p* = 0.041; *d* = 0.266) (Fig. 5). The control–massage interaction was significant for C|ShS|_L_ (*β*: 51%, 95% CI [34, 68]; *p* < 0.001; *d* = 1.082) and Max|ShS|_L_ (*β*: +0.9%, 95% CI [0.4, 1.4]; *p* = 0.001; *d* = 0.612) (Table 4).

**Table 4:** Linear mixed-effects model estimates of sequential within-subject effects of massage therapy on ultrasound outcomes (intention-to-treat analysis)

Parameter	Phase/interaction	Before LS mean [95% CI]	After LS mean [95% CI]	Adjusted difference estimate (*β*) LS mean [95% CI]	*p*-value	Effect size (*d*)
C|ShS|_L_ (%)	Control	213 [177, 250]	233 [196, 269]	19 [7, 31]	0.002	0.409
	Massage	233 [196, 269]	201 [164, 238]	-32 [-44, -19]	< 0.001	-0.673
	Control–massage*	-	-	51 [34, 68]	< 0.001	1.082
Max|ShS|_L_ (%)	Control	5.3 [4.5, 6.2]	5.7 [4.9, 6.6]	0.4 [0.02, 0.8]	0.041	0.266
	Massage	5.7 [4.9, 6.6]	5.2 [4.3, 6.1]	-0.5 [-0.9, -0.1]	0.010	-0.346
	Control–massage*	-	-	0.9 [0.4, 1.4]	0.001	0.612
1/α (unitless)	Control	0.71 [0.66, 0.76]	0.72 [0.67, 0.77]	0.01 [-0.02, 0.05]	0.449	0.098
	Massage	0.72 [0.67, 0.77]	0.70 [0.65, 0.75]	-0.02 [-0.06, 0.02]	0.273	-0.147
	Control–massage*	-	-	0.03 [-0.02, 0.08]	0.189	0.245
1/(κ + 1) (unitless)	Control	0.34 [0.33, 0.36]	0.33 [0.31, 0.34]	-0.02 [-0.04, 0.003]	0.089	-0.220
	Massage	0.33 [0.31, 0.34]	0.34 [0.33, 0.36]	0.01 [-0.01, 0.03]	0.153	0.192
	Control–massage*	-	-	-0.03 [-0.06, -0.003]	0.027	-0.412

Control–massage* represents the between-phase difference in adjusted changes (ΔΔLS mean), corresponding to the within-subject phase × time interaction

1/α = the reciprocal of the scatterer clustering parameter (scatterer density). 1/(κ + 1) = the diffuse-to-total signal power ratio parameter (scatterer organization)

*CI* Confidence interval, *C|ShS|*_*L*_ Cumulated absolute lateral shear strain magnitude, *d* Cohen’s d effect size, *LS* Least squares, *Max|ShS|*_*L*_ Maximum absolute lateral shear strain

#### Sensitivity analyses

Per-protocol analyses were consistent with the intention-to-treat results (Supplementary Tables 2–4). In a three-timepoint sensitivity analysis, the contrast comparing change during the massage phase with that during the waitlist phase [(T_2_–T_1_) *minus* (T_1_–T_0_)] remained highly significant for C|ShS|_L_ (estimate: -51; *p* < 0.001), with unchanged results after adjustment for visit order (*p* < 0.001) (Supplementary Table S5).

### Effects of treatments on the secondary outcomes

#### QUS HKD parameters (TLF microstructure)

In the randomized groups, no significant within-group or between-group effects were observed for the QUS HKD parameters (Table 3 and Supplementary Table S1). Similarly, in the within-subject massage analysis (Table 4), neither 1/α nor 1/(κ + 1) showed significant within-phase changes. However, a small but statistically significant control–massage interaction was detected for 1/(κ + 1) (*β* = -0.03, CI [-0.06, -0.003]; *p* = 0.027; *d* = -0.412), a finding that was also confirmed in the per-protocol analysis (Supplementary Table S4).

#### Pain and disability scores

Chiropractic was the only intervention associated with a clinically meaningful improvement in disability, as measured by the ODI score, compared with control (*p* = 0.041) (Table 5). No significant association between changes in C|ShS|_L_ and ODI scores (ρ = 0.261; *p* = 0.054) was observed.

**Table 5:** Proportion of participants reaching minimal clinically important differences by questionnaire and intervention

Questionnaire	MCID Status	Control (*n* = 20)	Massage (*n* = 18)	*p*-value (Massage *versus* Control)	Acupuncture (*n* = 19)	*p*-value (Acupuncture *versus* Control)	Chiropractic (*n* = 18)	*p*-value (Chiropractic *versus* Control)
BPI average pain	Yes (≥ 10%)	9 (45.0)	10 (55.6)	0.746	9 (47.4)	1.000	11 (61.1)	0.352
	No (< 10%)	11 (55.0)	8 (44.4)		10 (52.6)		7 (38.9)	
BPI interference	Yes (≥ 1)	2 (10.0)	4 (22.2)	0.395	6 (31.6)	0.127	7 (38.9)	0.058
	No (< 1)	18 (90.0)	14 (77.8)		13 (68.4)		11 (61.1)	
ODI	Yes (≥ 12%)	0 (0.0)	3 (16.7)	0.097	3 (15.8)	0.106	4 (22.2)	0.041
	No (< 12%)	20 (100.0)	15 (83.3)		16 (84.2)		14 (77.8)	
PROMIS Physical	Yes (≥ 3)	6 (30.0)	11 (61.1)	0.101	8 (42.1)	0.514	7 (38.9)	0.734
	No (< 3)	14 (70.0)	7 (38.9)		11 (57.9)		11 (61.1)	
PROMIS Mental	Yes (≥ 3)	4 (20.0)	4 (22.2)	1.000	7 (36.8)	0.301	6 (33.3)	0.468
	No (< 3)	16 (80.0)	14 (77.8)		12 (63.2)		12 (66.7)	

Numbers in parentheses are percentages that reflect the proportion of participants within each group who met or exceeded the established MCID threshold for each questionnaire outcome: ≥ 10% reduction for BPI average pain, ≥ 1-point reduction for BPI interference, ≥ 12% improvement for the Oswestry disability index (ODI), and ≥ 3-point improvement for PROMIS Physical and Mental T-scores. The *p*-values were calculated using Fisher’s exact test to compare each treatment group to the control group

*BPI* Brief pain inventory, *MCID* Minimal clinically important difference, *PROMIS* Patient-Reported Outcomes Measurement Information System

## Discussion

This study investigated the short-term effects of therapeutic interventions on TLF properties in participants with NSLBP, using a hybrid design. Participants were initially randomized into one of three parallel groups—acupuncture, chiropractic care, and waitlist control—for a 3-week intervention period. Subsequently, individuals from the control group received massage therapy, enabling a within-subject mechanistic responsiveness analysis. Ultrasound assessed TLF ShS and microstructural HKD metrics. In the randomized group analysis, chiropractic significantly reduced C|ShS|_L_ (*p* = 0.021; *d* = -0.322), while acupuncture elicited no detectable change (*p* = 0.130). C|ShS|_L_ worsened in the control group (*p* = 0.003; *d* = +0.387), and the chiropractic-control difference showed a moderate-to-large effect size (*p* = 0.001; *d* = -0.709). In the within-subject analysis, massage therapy induced a moderate-to-large reduction in C|ShS|_L_ (*p* < 0.001; *d* = -0.673), with a large interaction effect between the control and massage phases (*p* < 0.001; *d* = -1.082), indicating a substantial treatment-related change, confirmed by the sensitivity analysis.

Since 2010, interest in ultrasound imaging of the TLF has grown. A review by Pirri et al [44] found that most studies examined the TLF in the prone position, focusing on thickness, echogenicity, and structural and mechanical properties. However, methodological heterogeneity and low study quality limit the strength of the evidence. Diverse assessment techniques create challenges for standardization and comparison. For example, Brandl et al [45] quantified TLF deformation during seated trunk extension using the latissimus dorsi–TLF junction as a landmark. Liu et al [46] reported greater TLF and paraspinal muscle stiffness in NSLBP patients compared to controls using shear-wave elastography ultrasound. A recent meta-analysis by David et al [47] of 79 studies that employed elastography techniques—strain imaging, shear-wave imaging, and vibration sonoelastography—to evaluate back muscles’ biomechanics, showed that strain imaging offers good reliability, moderate discriminative validity, and moderate responsiveness to changes.

Regarding validation, the Lagrangian speckle model estimator is a robust, affine model-based strain technique that reduces noise amplification by avoiding displacement-field differentiation [48]. The method has demonstrated established reliability in vascular imaging [48], and in our previous work, it showed discriminative validity in differentiating NSLBP patients from healthy controls [28]. Similar ultrasound speckle-tracking approaches have also demonstrated excellent intra-rater reliability in other deformable tissues [49]. However, for TLF ShS, clinically meaningful thresholds (*e.g*., MCID or substantial clinical benefit), predictive validity, and responsiveness benchmarks remain undefined. Therefore, ShS should currently be regarded as a reproducible mechanistic imaging biomarker rather than an established clinical decision-making tool.

Our elastography technique quantified shear-induced angular deformation and shape changes within the TLF sublayers and the *erector spinae* aponeurosis. Mechanistically, elevated ShS values indicate abnormal TLF tension and disrupted interfascial sliding dynamics [28]. Supporting this interpretation, Brandl et al [50] observed irregular TLF deformation and inconsistent *erector spinae* muscle activation in individuals with NSLBP, suggesting impaired coordination between the muscle and fascia. In our prior case-control study [28], NSLBP participants exhibited significantly greater unadjusted C|ShS|_L_ mean ± standard deviation values than controls (327.1 ± 106.0% *versus* 290.2 ± 99.8%, *p* < 0.001), using the same imaging protocol. In the current trial, massage therapy reduced unadjusted C|ShS|_L_ values from a median [interquartile range] of 228% [149, 303] to 192% [134, 264]. Although baseline ShS values differed between cohorts, the consistent imaging approach allows cautious qualitative comparison. The magnitude of ShS reduction after massage approximates the earlier case-control difference. Although this comparison is limited by cohort differences and reliance on unadjusted values rather than model-based estimates, the large within-subject interaction supports a biomechanical response to massage therapy targeting the TLF and related fascial chains, distinct from natural progression or observation effects during the waitlist phase.

Few controlled studies have evaluated therapeutic effects on TLF biomechanics. Our previous trial found no immediate change in ShS after a brief massage session [28], likely due to limited intensity or duration. Vining et al observed sex-specific improvements in ShS after 8 weeks of chiropractic care using an ultrasound-displacement-based technique [21, 51]. Devantéry et al [24] found no immediate differences in TLF stiffness, as measured by shear-wave ultrasound, between real and simulated myofascial therapy, reinforcing the notion that short exposures may not induce detectable effects. Collectively, these results emphasize the importance of treatment duration and standardized elastography techniques in fascial research.

In this study, none of the active interventions produced statistically significant changes in the QUS HKD metrics. This absence of detectable microstructural adaptation over the 3 weeks should be interpreted with caution, as it may reflect biological limitations—such as insufficient intervention duration or intensity to induce collagen or extracellular matrix remodeling—or methodological factors, including the small sample size and the potential limited sensitivity of the HKD metrics to subtle structural changes.

Chiropractic was associated with a statistically significant improvement in ODI, with a higher proportion of participants meeting the MCID threshold than in controls (*p* = 0.041). An exploratory analysis showed no significant association between changes in ShS and ODI scores (Spearman ρ = 0.261, *p* = 0.054). These findings do not support a clear relationship between fascial mobility and clinical improvement in this sample.

This study has limitations. First, the heterogeneous nature of NSLBP may have led to the inclusion of participants with undetected specific pathologies, which could have influenced treatment response. Second, the trial was terminated early, enrolling 60 participants instead of the initially planned 96. Moreover, participants exhibited lower baseline ODI scores than expected in the original power calculation (median 17–24 *versus* a planned mean of 29). These factors likely reduced the study’s statistical power to detect between-group differences in clinical outcomes. Specifically, the smaller sample size increases the standard error, limiting the ability to detect moderate treatment effects. Additionally, enrolling a mildly disabled population narrows the potential range of improvement, creating a ceiling effect that further reduces the likelihood of detecting clinically meaningful changes. Together, these factors diminish the study’s sensitivity to detect significant effects on pain and disability outcomes. Furthermore, given the number of secondary endpoints and multiple group comparisons, these analyses should be considered exploratory, and their findings interpreted with caution. As such, the absence of statistical significance in these measures should not be overinterpreted. This trial is best viewed as a mechanistic investigation into fascial biomechanics, rather than a fully powered clinical efficacy study.

Three weekly sessions of chiropractic care or massage therapy, but not acupuncture, were associated with detectable biomechanical changes in the TLF in individuals with NSLBP. No microstructural changes were observed. Ultrasound measures showed significant reductions in TLF ShS, and chiropractic care was associated with modest improvement in disability. These findings indicate that ultrasound can detect short-term biomechanical responses, while the clinical implications require further investigation.

## Supplementary information

**Additional File 1:** **Table S1** Linear mixed-effects model estimates of between-group treatment differences in ultrasound outcomes (randomized parallel-group intention-to-treat analysis). **Table S2.** Linear mixed-effects model estimates of randomized parallel-group treatment effects on ultrasound outcomes (per-protocol analysis). **Table S3.** Linear mixed-effects model estimates of between-group treatment differences in ultrasound outcomes (randomized parallel-group per-protocol analysis). **Table S4.** Linear mixed-effects model estimates of sequential within-subject effects of massage therapy on ultrasound outcomes (per-protocol analysis). **Table S5.** Sensitivity analysis of the sequential within-subject phase using a three-time point linear mixed-effects model with adjustment for visit order (intention-to-treat analysis).

## Data Availability

The datasets used and/or analyzed during the current study are available from the corresponding author on reasonable request.

## Abbreviations

1/(*κ* + 1): Diffuse-to-total signal power ratio (scatterer organization)
1/*α*: Reciprocal of the scatterer clustering parameter (scatterer density)
BPI: Brief pain inventory
C|ShS|_L_: Cumulated absolute lateral shear strain magnitude
CI: Confidence interval
HKD: Homodyned K-distribution
Max|ShS|_L_: Maximum absolute lateral shear strain
MCID: Minimal clinically important difference
NSLBP: Nonspecific low back pain
ODI: Oswestry disability index
PROMIS: Patient-Reported Outcomes Measurement Information System
QUS: Quantitative ultrasound
ROI: Region of interest
ShS: Shear strain
TLF: Thoracolumbar fascia
