# Supplementary material for: Quantitative ultrasound evaluation of the thoracolumbar fascia after manual and acupuncture therapies: an exploratory mechanistic randomized controlled trial with a sequential within-subject phase
Source: Eur Radiol Exp. 2026 Jun 19;10:94. doi: 10.1186/s41747-026-00754-7 (PMC13282428; doi:10.1186/s41747-026-00754-7)
Supplement: Supplementary file 1 — Additional File 1: Table S1 Linear mixed-effects model estimates of between-group treatment differences in ultrasound outcomes (randomized parallel-group intention-to-treat analysis). Table S2. Linear mixed-effects model estimates of randomized parallel-group treatment effects on ultrasound outcomes (per-protocol analysis). Table S3. Linear mixed-effects model estimates of between-group treatment differences in ultrasound outcomes (randomized parallel-group per-protocol analysis). Table S4. Linear mixed-effects model estimates of sequential within-subject effects of massage therapy on ultrasound outcomes (per-protocol analysis). Table S5. Sensitivity analysis of the sequential within-subject phase using a three-time point linear mixed-effects model with adjustment for visit order (intention-to-treat analysis). [file 41747_2026_754_MOESM1_ESM.pdf]

Quantitative ultrasound evaluation of the thoracolumbar fascia after manual and acupuncture therapies: an exploratory mechanistic randomized controlled trial with a sequential within-subject phase

ELECTRONIC SUPPLEMENTARY MATERIAL

Treatment protocols

This document describes the specific protocols for the three therapeutic interventions.

Acupuncture. The goal is to reestablish balanced tissue, neural, autonomic, and constitutional function, as summarised below.

1. Reduce myofascial and segmental tension in the lumbosacral region to improve fascial gliding, relieve local stagnation, and decrease nociceptive signaling.
2. Restore interlayer fascial mobility, targeting zones of densification and restricted movement through a combination of local and distal needling techniques informed by palpation and observation.
3. Modulate central sensitization by stimulating neuroregulatory acupuncture points associated with descending pain inhibitory pathways, thereby reducing chronic pain perception and enhancing endogenous analgesic mechanisms.
4. Regulate autonomic function and address emotional contributors, including stress, sleep disturbances, and irritability, that exacerbate pain persistence.
5. Tonify underlying deficiencies frequently observed in chronic pain presentations, such as depletion of Kidney and Spleen Qi, to restore constitutional resilience and support systemic recovery.
6. Invigorate Qi and Blood circulation and dredge obstructed channels traversing the lower back, particularly the Urinary Bladder and Kidney meridians, to alleviate pain, restore movement, and reestablish harmonious flow through affected tissues.
7.

Duration:

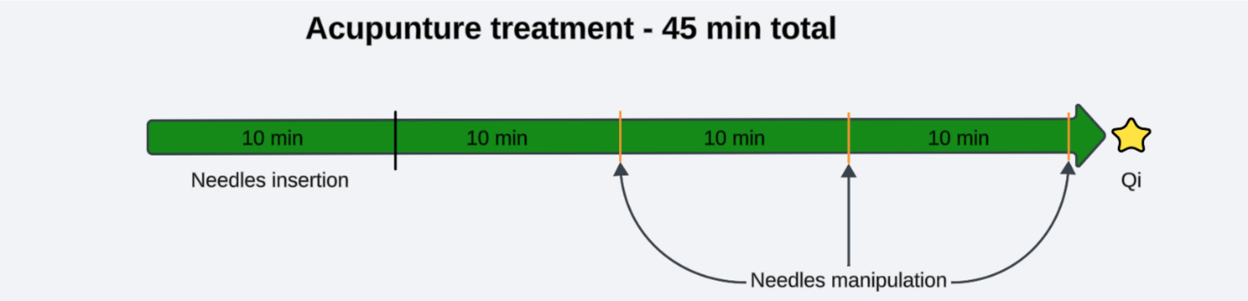

Detailed protocol:

Acupuncture points	Description
Jia ji from L1 to L5	Jia ji from L1 to L5, 0.5 cun on each side of the lower border of the spinous processes. (A cun is a traditional Chinese unit of length, it is equivalent to the width of the thumb at the knuckle.)
Shu points of the kidney and large intestine	1.5 cun on each side of the lower border of spinous processes of L2 and L4. Shen shu and da chang shu.
Yao yan, in the region of the waist	In the depression in the lower border of L4, 3.5 cun on each side of the posterior midline.
Ju liao GB29	Located at the midpoint of the line connecting the anterior superior iliac spine and the prominence of the greater trochanter of the femur.
Huan tiao GB30	At the junction between the lateral 1/3 and the medial 2/3 of the line that links the greater trochanter prominence and the hiatus of the sacrum.
Feng shi GB31	On the midline of the lateral side of the thigh, 7 cun above the transverse popliteal crease.
Wei Zhong UB40	On midpoint of the transverse crease of the popliteal fossa, between the femoral biceps muscle and semitendinosus muscle.
Cheng shan UB57	In the center of the posterior side of the lower leg between the bellies of the gastrocnemius muscles.
Zhi Bian UB54	Located in the region of the buttocks, at same level as the 4th posterior sacral foramens, 3 cun lateral the middle sacral crest.
Kun lun UB60	Located on the posterior side to the external malleolus in the depression between the prominence of the external malleolus and achilles tendon.
Ashi points	Ashi points will be used if sensitive or tender upon palpation by the acupuncturist.

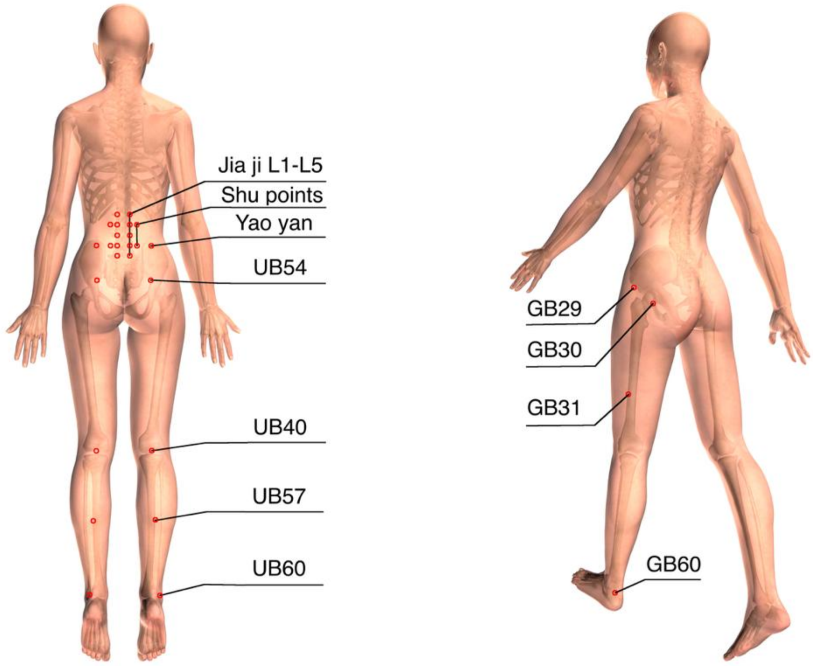
Jia ji L1-L5
Shu points
Yao yan
UB54
UB40
UB57
UB60
GB29
GB30
GB31
GB60

Multimodal chiropractic care. The goal is to reduce tension in the paraspinal muscles, quadratus lumborum, iliopsoas, diaphragm, gluteal and hamstrings muscles, and to improve mobility of each vertebra and sacroiliac joints.

Duration:

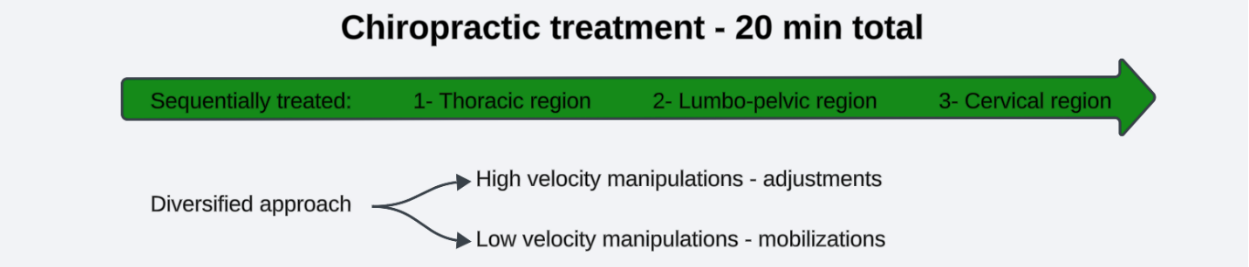

Detailed protocol:

Technique	Description
Spine and soft tissue palpation in the prone position	The patient is prone. The clinician initially palpates the posterior musculature of the back and pelvis with the heel of the hand. The theraspist then segmentally assesses the anteroposterior movement of the spine.<br><br>Next, the chiropractor palpates the intervertebral segments more specifically with the tips of the thumbs, using the lightest possible pressure to assess the rotational movement of the vertebrae.<br><br>With the patient's verbal consent, the position of the coccyx is also palpated.<br><br>Length: 30s
Visual check of apparent leg length	With the patient in a prone position, gently pull on the patient's lower limbs to balance the relaxed patient's pelvis.<br><br>First with knees in extension, then at 90° flexion, assess the difference in leg length from the edge of the shoe heel.<br><br>The pattern of variation indicates the side and direction of joint blockage in need of correction.<br><br>Length: 2.5 min
Massage with vibrator	The clinician places the vibrator on the patient's upper back and moves it caudally along the spine. At the thoracolumbar level, the vibrator is rotated 90° to cover the lumbar region. Slide over the buttocks from medial to lateral, then over the lateral aspect of the thigh. Repeat on opposite side. Cover the posterior aspect of the thighs and slide over the calves.<br><br>Length: 30–45s
Treatment of posterior soft tissue (stretching and myofascial	Application of constant ischemic deep pressure on trigger points present during initial palpation, to promote release of muscle tension in the paraspinals, quadratus lumborum and gluteus maximus.<br><br>Passive stretching of piriformis and hamstrings with muscle compression. Compression of the proximal tendon attachment of

trigger point therapy)	the piriformis(s) in a shortened position: ipsilateral knee flexed, with the femur in external rotation. The clinician stretches the femur in internal rotation, holding the ankle with the caudal hand while maintaining compression on the piriformis. The stretch is repeated 3 times. Can also be performed with compression in the muscle belly.<br><br>Compression of proximal hamstrings in shortened position: knee bent. The clinician guides knee extension while maintaining compression on the hamstring. The stretch is repeated 3 times.<br><br>Length: 2 min
Adjustment of thoracolumbar intervertebral segments and/or sacroiliac joints	Manipulation of inter-articular segments noted during initial palpation. With the diversified chiropractic technique, with the patient in lateral decubitus, the clinician facilitates movement of the vertebra by maintaining contact with the spinous process and causing a rotational movement of the patient's lower body, in the appropriate direction and with the amount of force adapted to the severity of the blockage or the patient's level of tolerance. The lumbar roll technique can also be used.<br><br>Manual (external) chiropractic adjustment of coccyx if noted in anterior position on initial palpation.<br><br>Manual mobilization of the hypomobile sacroiliac joint according to the pattern observed on palpation and assessment of leg length.<br><br>Check the mobility of each segment after each procedure to ensure successful mobilization.<br><br>Reassess the lower limb length.<br><br>Length: 2 min
Treatment of anterior facial soft tissue in supine position using trigger point therapy	Application of constant ischemic deep pressure with the thumbs on the trigger points present when palpating the psoas iliacus and diaphragm.<br><br>Length: 30–45 s

Massage. The goal is to release tension in multiple fascial chains connected to the lower back, as palpated by the therapist.

Duration:

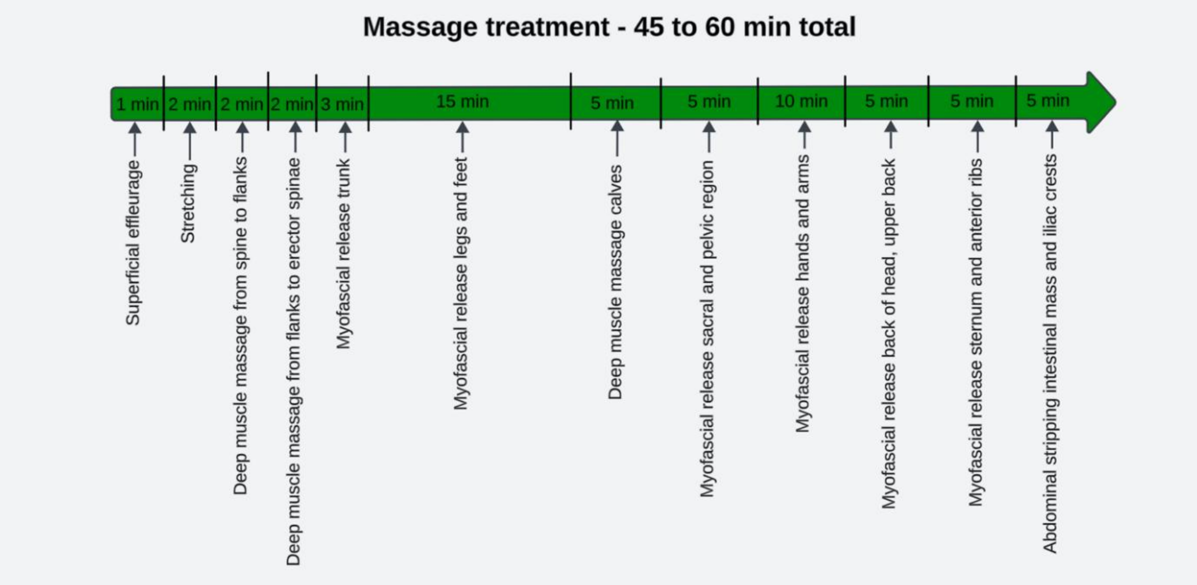

Detailed protocol:

1. Effleurage using palm of the hand 1m

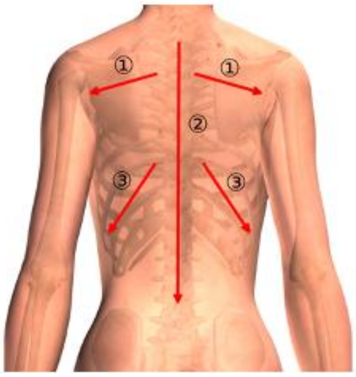

2. Stretching 2m

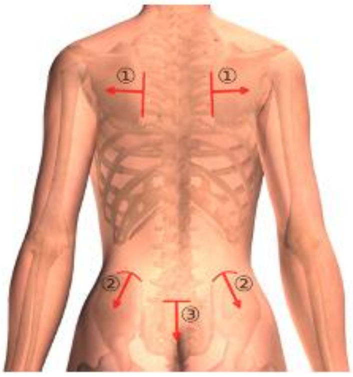

3. Muscle stripping
(from spine towards the outside by maintaining pressure with one thumb at the spinous process 2m

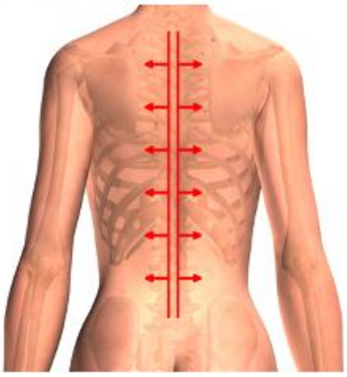

4. Muscle stripping
(from outside elector spinae towards the inside by maintaining pressure with one thumb) 2m

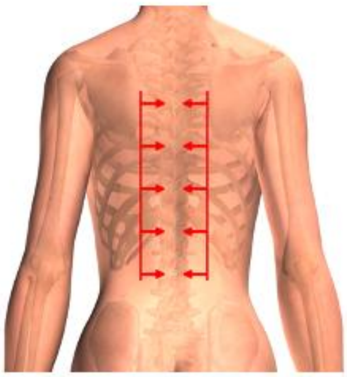

5. Fascial release
(Stimulate by nail along scapula and libs and slide the area laterally for recovering mobility of deep fascia) 3m

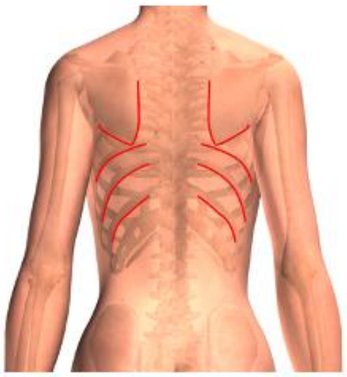

6. Fascial release
(Stimulate along bones by nail, phalanges, metatarsal 1st 5th, calcaneus, tibia, fibula for recovering mobility of deep fascia) 15m

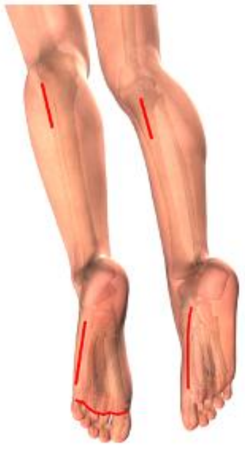

7. Muscle stripping
(Stimulate along calf by nail and move the entire calf laterally for loosen multi layered adhesions) 5m

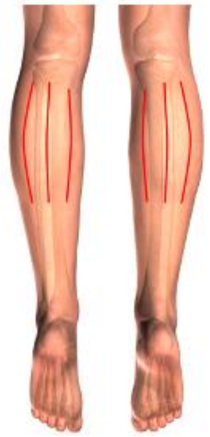

8. Fascial release
(Stimulate dorsal sacral foramina, Sacro-iliac joint, iliac crest, femoral head by nail for recovering mobility of deep fascia) 5m

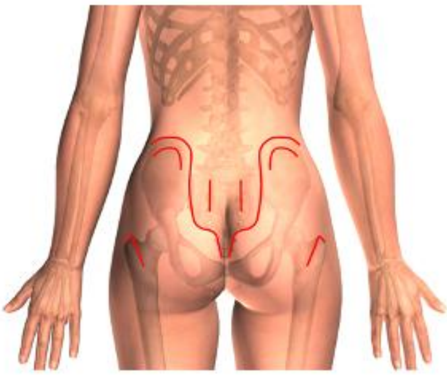

9. Fascial release
(Stimulate subspinous scapula, proximal upper carpal bone, proximal radius, distal radius, distal ulna, proximal phalanges, root bone of hand with the therapist's nails to recover the mobility of the deep fascia) 10m

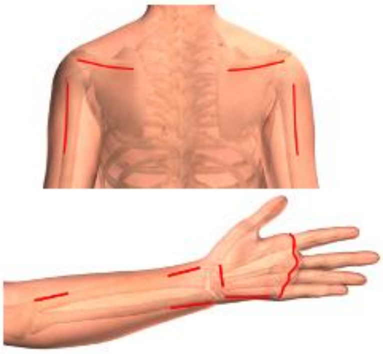

10. Fascial release
(Stimulate all over the participants back head with the therapist's nails to recover the mobility of the deep fascia) 5m

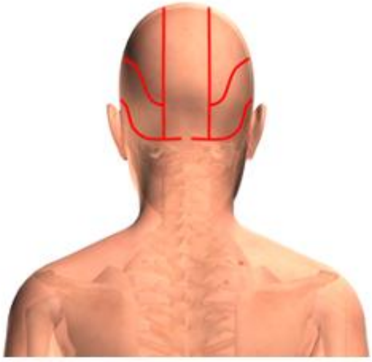

11. Fascial release
(Stimulate Subclavian, upper carpal bones, rib joints, sternal body with the therapist's nails to recover the mobility of the deep fascia) 5m

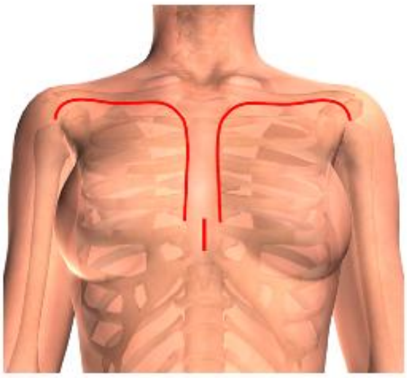

12. Abdominal stripping
(Move the small intestinal mass from the outside to the center with the fingers. Shift and loosen fascial adhesions on the iliac crest in a central direction) 5m

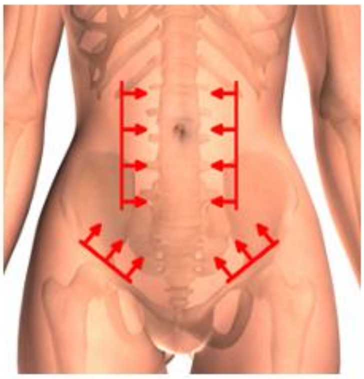

Supplementary Tables

Table S1 Linear mixed-effects model estimates of between-group treatment differences in ultrasound outcomes (randomized parallel-group intention-to-treat analysis)

Parameter	Group pairs	Adjusted difference estimate (*β*) LS mean [95% CI]	*p*-value	Effect size (*d*)
C|ShS|L (%)	Control – Acupuncture	29 [7, 51]	0.005	0.591
	Control – Chiropractic	35 [12, 57]	0.001	0.709
	Acupuncture – Chiropractic	6 [–17, 28]	1.000	0.118
Max|ShS|L (%)	Control – Acupuncture	0.6 [–0.1, 1.2]	0.081	0.414
	Control – Chiropractic	0.6 [–0.04, 1.3]	0.076	0.425
	Acupuncture – Chiropractic	0.02 [–0.7, 0.7]	1.000	0.011
1/α (unitless)	Control – Acupuncture	0.02 [–0.04, 0.09]	1.000	0.171
	Control – Chiropractic	–0.02 [–0.08, 0.05]	1.000	–0.105
	Acupuncture – Chiropractic	–0.04 [–0.11, 0.03]	0.434	–0.276
1/(κ+1) (unitless)	Control – Acupuncture	–0.01 [–0.04, 0.02]	0.886	–0.193
	Control – Chiropractic	–0.02 [–0.05, 0.01]	0.277	–0.314
	Acupuncture – Chiropractic	–0.01 [–0.04, 0.02]	1.000	–0.121

CI Confidence interval, *C|ShS|L* Cumulated absolute lateral shear strain magnitude, *d* Cohen effect size. *LS* Least squares mean, *Max|ShS|L* Maximum absolute lateral shear strain. 1/α = the reciprocal of the scatterer clustering parameter (scatterer density). 1/(κ+1) = the diffuse-to-total signal power ratio parameter (scatterer organization).

Table S2 Linear mixed-effects model estimates of randomized parallel-group treatment effects on ultrasound outcomes (per-protocol analysis)

Parameter	Group	Before LS mean [95% CI]	After LS mean [95% CI]	Adjusted change estimate (*β*) [95% CI]	*p*-value	Effect size (*d*)
C|ShS|L (%)	Control	218 [187, 249]	237 [206, 268]	19 [7, 32]	0.003	0.390
	Acupuncture	221 [189, 252]	211 [179, 243]	–10 [–23, 3]	0.149	–0.194
	Chiropractic	223 [190, 256]	208 [175, 241]	–15 [–29, –1]	0.030	–0.303
Max|ShS|L (%)	Control	5.3 [4.5, 6.0]	5.7 [4.9, 6.4]	0.4 [0.02, 0.8]	0.039	0.268
	Acupuncture	5.5 [4.7, 6.3]	5.3 [4.5, 6.1]	–0.2 [–0.6, 0.2]	0.252	–0.154
	Chiropractic	5.5 [4.7, 6.3]	5.3 [4.5, 6.1]	–0.2 [–0.6, 0.2]	0.276	–0.152
1/α (unitless)	Control	0.73 [0.69, 0.78]	0.75 [0.71, 0.79]	0.01 [–0.02, 0.05]	0.442	0.100
	Acupuncture	0.73 [0.69, 0.78]	0.72 [0.68, 0.77]	–0.01 [–0.05, 0.03]	0.649	–0.061
	Chiropractic	0.73 [0.68, 0.77]	0.76 [0.71, 0.8]	0.03 [–0.01, 0.07]	0.141	0.201
1/(κ+1) (unitless)	Control	0.34 [0.33, 0.36]	0.33 [0.31, 0.34]	–0.02 [–0.04, –0.0002]	0.047	–0.258
	Acupuncture	0.34 [0.33, 0.36]	0.34 [0.33, 0.35]	–0.004 [–0.02, 0.01]	0.707	–0.050
	Chiropractic	0.33 [0.31, 0.34]	0.33 [0.32, 0.35]	0.01 [–0.01, 0.02]	0.584	0.075

CI Confidence interval, *C|ShS|L* Cumulated absolute lateral shear strain magnitude, *d* Cohen effect size, *LS* Least squares mean, *Max|ShS|L* Maximum absolute lateral shear strain, *β* = percentage change relative to the reference group mean. 1/α = the reciprocal of the scatterer clustering parameter (scatterer density). 1/(κ+1) = the diffuse-to-total signal power ratio parameter (scatterer organization).

Table S3 Linear mixed-effects model estimates of between-group treatment differences in ultrasound outcomes (randomized parallel-group per-protocol analysis)

Parameter	Group pairs	Adjusted difference estimate (*β*) LS mean [95% CI]	*p*-value	Effect size (*d*)
$C\|ShS\|_L$ (%)	Control – Acupuncture	29 [7, 51]	0.006	0.585
	Control – Chiropractic	34 [12, 57]	0.001	0.693
	Acupuncture – Chiropractic	5 [–17, 28]	1.000	0.108
$Max\|ShS\|_L$ (%)	Control – Acupuncture	0.6 [–0.04, 1.3]	0.072	0.423
	Control – Chiropractic	0.6 [–0.05, 1.3]	0.082	0.420
	Acupuncture – Chiropractic	0.004 [–0.7, 0.7]	1.000	–0.003
1/α (unitless)	Control – Acupuncture	0.02 [–0.04, 0.09]	1.000	0.160
	Control – Chiropractic	–0.01 [–0.08, 0.05]	1.000	–0.101
	Acupuncture – Chiropractic	–0.04 [–0.1, 0.03]	0.508	–0.262
1/(κ+1) (unitless)	Control – Acupuncture	–0.01 [–0.05, 0.02]	0.788	–0.208
	Control – Chiropractic	-0.02 [–0.06, 0.01]	0.233	–0.333
	Acupuncture – Chiropractic	–0.01 [–0.04, 0.02]	1.000	–0.125

CI Confidence interval, $C|ShS|_L$ Cumulated absolute lateral shear strain magnitude, *d* Cohen effect size, *LS* Least squares mean, $Max|ShS|_L$ Maximum absolute lateral shear strain. 1/α = the reciprocal of the scatterer clustering parameter (scatterer density). 1/(κ+1) = the diffuse-to-total signal power ratio parameter (scatterer organization).

Table S4 Linear mixed-effects model estimates of sequential within-subject effects of massage therapy on ultrasound outcomes (per-protocol analysis)

Parameter	Phase / Interaction	Before LS mean [95% CI]	After LS mean [95% CI]	Adjusted Difference Estimate (*β*) LS mean [95% CI]	*p*-value	Effect size (*d*)
C|ShS|$_L$ (%)	Control	213 [176, 250]	233 [196, 269]	20 [7, 32]	0.002	0.413
	Massage	232 [195, 268]	200 [163, 237]	–31 [–44, –18]	< 0.001	–0.659
	Control – Massage*	-	-	51 [33, 68]	< 0.001	1.072
Max|ShS|$_L$ (%)	Control	5.3 [4.5, 6.2]	5.7 [4.9, 6.6]	0.4 [0.03, 0.8]	0.036	0.274
	Massage	5.7 [4.9, 6.6]	5.2 [4.3, 6.1]	–0.5 [–0.9, –0.1]	0.010	–0.352
	Control – Massage*	-	-	0.9 [0.4, 1.4]	0.001	0.627
1/α (unitless)	Control	0.71 [0.66, 0.76]	0.72 [0.67, 0.77]	0.01 [–0.02, 0.05]	0.417	0.105
	Massage	0.72 [0.67, 0.77]	0.7 [0.65, 0.75]	–0.02 [–0.05, 0.02]	0.365	–0.124
	Control – Massage*	-	-	0.03 [–0.02, 0.08]	0.224	0.229
1/(κ+1) (unitless)	Control	0.34 [0.33, 0.36]	0.33 [0.31, 0.34]	–0.02 [–0.04, 0.001]	0.065	–0.24
	Massage	0.33 [0.31, 0.34]	0.34 [0.33, 0.36]	0.01 [–0.01, 0.04]	0.148	0.198
	Control – Massage*	-	-	–0.03 [–0.06, –0.01]	0.021	–0.437

Control – Massage* represents the between-phase difference in adjusted changes (ΔΔLS mean), corresponding to the within-subject phase × time interaction. *CI* Confidence interval, *C|ShS|$_L$* Cumulated absolute lateral shear strain magnitude, *d* Cohen effect size, *LS* Least squares mean, *Max|ShS|$_L$* Maximum absolute lateral shear strain. 1/α = the reciprocal of the scatterer clustering parameter (scatterer density). 1/(κ+1) = the diffuse-to-total signal power ratio parameter (scatterer organization).

Table S5. Sensitivity analysis of the sequential within-subject phase using a three-timepoint linear mixed-effects model with adjustment for visit order (intention-to-treat analysis)

Parameter	Group pairs	Adjusted difference estimate (*β*) LS mean [95% CI]	*p*-value	Effect size (*d*)
C|ShS|$_L$ (%)	Massage – Control	–51 [–73, –30]	< 0.001	–1.082
Max|ShS|$_L$ (%)	Massage – Control	–0.88 [–1.52, –0.24]	0.007	–0.612
1/α (unitless)	Massage – Control	–0.03 [–0.10, 0.03]	0.288	–0.245
1/(κ+1) (unitless)	Massage – Control	0.03 [–0.004, 0.07]	0.086	0.412

CI Confidence interval, *C*|*ShS*|$_L$ Cumulated absolute lateral shear strain magnitude, *d* Cohen effect size, *LS* Least squares mean, *Max*|*ShS*|$_L$ Maximum absolute lateral shear strain. 1/α = the reciprocal of the scatterer clustering parameter (scatterer density). 1/(κ+1) = the diffuse-to-total signal power ratio parameter (scatterer organization).
